# Notes and News

**Published:** 1891-10-10

**Authors:** 


### Notes and News.

Collected and Communicated.

An amusing difficulty is occupying the minds of the Relieving Officers of the Camberwell division and others. It appears that a certain number of voters have been diensfranchised on the plea that they had received parochial relief. On the matter being inquired into, it was found that the relief was administered in the form of beef tea! The revising barrister is taking time to consider whether beef tea, as supplied by the parish can be regarded as a disenfranchising fluid or otherwise?

The Queenstown Dispensary Election is now a thing of the past, and Dr. Hodges has been duly elected. His success was celebrated by his friends by the lighting of tar barrels in the vicinity of his residence. Notwithstanding that the election is a *fait accompli*, tongues and pens of Protestants and Catholics are still busy abusing one another. Committee meetings are still disorderly and disgraceful, and a paragraph appears in a Cork paper headed "Cowards and Traitors, Stand Aside." Cork papers will be quite at a loss when this exciting topic, which has occupied their pages for so long, is laid to rest.

The *City Press*, whilst regretting that dissension is so constantly breaking out amongst the active members of the Hospital Saturday Fund, says thanks in a great measure to the new vigour infused into the fund by Sir James Whitehead, the originator of the penny-a-week movement, the collection in the workshops of the metropolis has shown a steady improvement during the past few years, although the sum raised still falls far short of the amount that was at one time spoken of. Despite the fact that the increased expenditure determined upon has answered its purpose, and proved the means of increasing the annual income by no less than 20 per cent., not a few of the delegates appear to consider that the outlay has not been productive of sufficient fruit. As far as the present is concerned, such an objection is, perhaps, worthy of consideration, seeing that the fund has not progressed side by side with the expenditure. On the other hand it must not be forgotten that the Committee are working for the future, and that for a few years, at any rate, they can hardly expect to receive the full benefit of the expenditure they have incurred in connection with the onward movement. Apparently the delegates, as a body, are a little doubtful as to the wisdom of casting the bread upon the waters in the hope that it will return to them after many days. Under the circumstances the committee will do well to wait for a few years before they consider the advisability of in some way drawing in their horns as regards the expenditure. Three or four years hence they will be able to judge far better than they can at present whether the new departure is proving the gold mine its instigators still believe it to be.

Mr. Albert Napper, founder of cottage hospitals, celebrated his golden wedding at Guildford on September 23rd, when over 200 friends from different parts of the country attended to personally offer their congratulations. Mr. Napper commenced practice at Guildford in 1839, being the fifth member of his family who had taken up the profession of a surgeon. He retired in 1881, but still takes the greatest interest in all hospital matters, and is on the Committee of the Nurses' Co-operation. His son is a surgeon at Cranleigh, his daughter is Matron of the Surrey Convalescent Home at Seaford.

Two cases of human skin shedding are reported from the United States. According to a paper by Dr. I. Frank, recently read before the Chicago Medical Society, one of the subjects is a man who has obtained a new skin every year of his life. The shedding occurs in July, and is accompanied by feverish tremors which last for twelve hours. The old skin turns red and peels off, leaving a soft rosy skin underneath, which is so tender at first that he is obliged to wear soft gloves and mocassins for a week. The other case is that of a lady, 39 years old, who ever since 1876 has renewed her epidermis every second or third year.

The Birmingham Hospital Saturday Fund are to be congratulated on the prompt and business-like manner in which they have set about procuring the proposed Convalescent Home in connection with the fund. At the special meeting, held last week, a detailed scheme was laid before the public. By a careful comparison of the expenditures of similar institutions in the Kingdom, it has been decided that an income of £1,800 will cover the whole cost of maintenance at the new home. It is estimated that forty patients will be maintained at an average expense of £2 per head. This sum would be actually largely reduced by payments received, which have not been taken into account in the present estimate. The committee have been fortunate in finding promising premises, and land ready to hand, in the form of a mansion, standing in twenty acres of ground, near Llandudno, North Wales. A well-stocked garden and farm buildings form part of the property, and comparatively little alteration will be required to the house itself. It is probable that the choice of the committee will meet with general approval. The only complaint so far that has been made is that the locality is too aristocratic! We do not think fears need be entertained even by the most socialistic on this score.

The Homerton Hospital scandal has given a portion of the press and the public an opportunity to throw stones at the hospital system in general. They state that the confidence of the public in hospital relief as at present carried out is fast waning, and that proper official control is the only remedy for existing defects of management. Now, we beg to differ entirely from this gloomy view of the situation, and congratulate the hospitals and the public that an influence so potent as public opinion has now fairly taken the helm in hospital matters. Thereby, doubtless, many a long-standing scandal will come to light, and also be duly removed. We also deny entirely the assertion of one paper that "although essentially public institutions, the public has no voice and no shadow of control over their administration." First of all, we revert to the important factor, public opinion, which no hospital dependent on public contributions can possibly ignore; and secondly, we beg to remind our contemporary of the representative nature of the governing bodies of most of the larger hospitals. Finally, we deny that the hospital system would be benefited by being controlled by a "separate elected central Board," charged (as it is aptly put) expressly and solely with the function of keeping the hospitals straight.

